# Treatments, resource utilization, and outcomes of COVID-19 patients presenting to emergency departments across pandemic waves: an observational study by the Canadian COVID-19 Emergency Department Rapid Response Network (CCEDRRN)

**DOI:** 10.1007/s43678-022-00275-3

**Published:** 2022-04-01

**Authors:** Corinne M. Hohl, Rhonda J. Rosychuk, Jeffrey P. Hau, Jake Hayward, Megan Landes, Justin W. Yan, Daniel K. Ting, Michelle Welsford, Patrick M. Archambault, Eric Mercier, Kavish Chandra, Philip Davis, Samuel Vaillancourt, Murdoch Leeies, Serena Small, Laurie J. Morrison, the Canadian COVID-19 Rapid Response Network (CCEDRRN) investigators for the Network of Canadian Emergency Researchers, for the Canadian Critical Care Trials Group

**Affiliations:** 1 Department of Emergency Medicine, University of British Columbia, Vancouver, BC Canada; 2 Centre for Clinical Epidemiology and Evaluation, Vancouver Coastal Health Research Institute, Vancouver, BC Canada; 3 Department of Pediatrics, University of Alberta, Edmonton, AB Canada; 4 Department of Emergency Medicine, University of Alberta, Edmonton, AB Canada; 5 Division of Emergency Medicine, University of Toronto, Toronto, ON Canada; 6 University Health Network, Toronto, ON Canada; 7 Division of Emergency Medicine, London Health Sciences Centre, London, ON Canada; 8 Schulich School of Medicine and Dentistry, Western University, London, ON Canada; 9 Division of Emergency Medicine, McMaster University, Hamilton, ON Canada; 10 Hamilton Health Sciences, Hamilton, ON Canada; 11 Department of Family Medicine and Emergency Medicine, Université Laval, Quebec, QC Canada; 12 Centre de recherche du Centre intégré de santé et de services sociaux de Chaudière-Appalaches, Levis, QC Canada; 13 Centre de Recherche, CHU de Québec, Université Laval, Quebec, QC Canada; 14 VITAM (Centre de recherche en santé durable), Quebec, QC, Canada; 15 Department of Emergency Medicine, Dalhousie Medicine New Brunswick, Saint John, NB Canada; 16 Department of Emergency Medicine, Saint John Regional Hospital, Saint John, NB Canada; 17 Department of Emergency Medicine, University of Saskatchewan, Saskatoon, SK Canada; 18 Department of Emergency Medicine, St Michael’s Hospital, Unity Health Toronto, Toronto, ON Canada; 19 Department of Emergency Medicine, University of Manitoba, Winnipeg, MB Canada; 20 Section of Critical Care Medicine, University of Manitoba, Winnipeg, MB Canada

**Keywords:** COVID-19; coronavirus disease 2019; SARS-COV-2; resource utilization; patient outcomes; pandemic waves

## Abstract

**Background:**

Treatment for coronavirus disease 2019 (COVID-19) evolved between pandemic waves. Our objective was to compare treatments, acute care utilization, and outcomes of COVID-19 patients presenting to emergency departments (ED) across pandemic waves.

**Methods:**

This observational study enrolled consecutive eligible COVID-19 patients presenting to 46 EDs participating in the Canadian COVID-19 ED Rapid Response Network (CCEDRRN) between March 1 and December 31, 2020. We collected data by retrospective chart review. Our primary outcome was in-hospital mortality. Secondary outcomes included treatments, hospital and ICU admissions, ED revisits and readmissions. Logistic regression modeling assessed the impact of pandemic wave on outcomes.

**Results:**

We enrolled 9,967 patients in 8 provinces, 3,336 from the first and 6,631 from the second wave. Patients in the second wave were younger, fewer met criteria for severe COVID-19, and more were discharged from the ED. Adjusted for patient characteristics and disease severity, steroid use increased (odds ratio [OR] 7.4; 95% confidence interval [CI] 6.2–8.9), and invasive mechanical ventilation decreased (OR 0.5; 95% CI 0.4–0.7) in the second wave compared to the first. After adjusting for differences in patient characteristics and disease severity, the odds of hospitalization (OR 0.7; 95% CI 0.6–0.8) and critical care admission (OR 0.7; 95% CI 0.6–0.9) decreased, while mortality remained unchanged (OR 0.7; 95% CI 0.5–1.1).

**Interpretation:**

In patients presenting to cute care facilities, we observed rapid uptake of evidence-based therapies and less use of experimental therapies in the second wave. We observed increased rates of ED discharges and lower hospital and critical care resource use over time. Substantial reductions in mechanical ventilation were not associated with increasing mortality. Advances in treatment strategies created health system efficiencies without compromising patient outcomes.

**Trial registration:**

Clinicaltrials.gov, NCT04702945.

**Supplementary Information:**

The online version contains supplementary material available at 10.1007/s43678-022-00275-3.

## Résumé

**Contexte:**

Le traitement de la maladie à coronavirus 2019 (COVID-19) a évolué entre les vagues pandémiques. Notre objectif était de comparer les traitements, l'utilisation des soins aigus et les résultats des patients atteints de la maladie COVID-19 se présentant aux urgences à travers les vagues de pandémie.

**Méthodes:**

Cette étude observationnelle a recruté des patients COVID-19 éligibles consécutifs se présentant à 46 services d'urgence participant au Réseau canadien de réponse rapide aux services d'urgence COVID-19 (CCEDRRN) entre le 1er mars et le 31 décembre 2020. Nous avons recueilli des données au moyen d’un examen rétrospectif des dossiers. Notre principal résultat a été la mortalité à l’hôpital. Les résultats secondaires incluaient les traitements, les admissions à l'hôpital et aux soins intensifs, les revisites aux urgences et les réadmissions. La modélisation par régression logistique a évalué l'impact de la vague de pandémie sur les résultats.

**Résultats:**

Nous avons recruté 9 967 patients dans 8 provinces, 3 336 de la première vague et 6 631 de la deuxième vague. Les patients de la deuxième vague étaient plus jeunes, moins nombreux à répondre aux critères de gravité de la COVID-19 et plus nombreux à quitter les urgences. Après ajustement en fonction des caractéristiques des patients et de la gravité de la maladie, le recours aux stéroïdes a augmenté (rapport de cotes [RC] 7.4 ; intervalle de confiance à 95 % [IC] 6.2–8.9) et la ventilation mécanique invasive a diminué (RC 0.5 ; IC à 95 % 0.4–0.7) lors de la deuxième vague par rapport à la première. Après ajustement pour tenir compte des différences dans les caractéristiques des patients et la gravité de la maladie, les probabilités d'hospitalisation (RC 0.7 ; IC à 95 % 0.6–0.8) et d'admission en soins intensifs (RC 0.7 ; IC à 95 % 0.6–0.9) ont diminué, tandis que la mortalité est restée inchangée (RC 0.7 ; IC à 95 % 0.5–1.1).

**Interprétation:**

Chez les patients se présentant dans les établissements de soins de santé, nous avons observé une adoption rapide des thérapies fondées sur des données probantes et un moindre recours aux thérapies expérimentales lors de la deuxième vague. Nous avons observé une augmentation des taux de sortie des services d'urgence et une diminution de l'utilisation des ressources hospitalières et des soins intensifs au fil du temps. Les réductions substantielles de la ventilation mécanique n'étaient pas associées à une augmentation de la mortalité. Les progrès réalisés dans les stratégies de traitement ont permis d'améliorer l'efficacité des systèmes de santé sans compromettre les résultats pour les patients.

## Clinician’s capsule

**Table:** 

***What is known about the topic?***
The patient population affected by and treatments for coronavirus disease 2019 (COVID-19) changed over the course of the pandemic.
***What did this study ask?***
How did treatments, hospital utilization and patient outcomes compare between the first two pandemic waves?
***What did this study find?***
We observed more steroid use, and less mechanical ventilation and critical care utilization with stable mortality during the second wave.
***Why does this study matter to clinicians?***
This study provides real-world evidence that practice changes in the second wave were safe and associated with lower resource utilization.

## Introduction

COVID-19 continues to place a strain on acute care hospitals. Early reports from the first wave of the pandemic were critical in allowing clinicians to gain an understanding of a new disease entity [1–6], but reflected convenience samples of patients with more severe disease and typical presentations due to limited testing capacity [7]. Most studies omitted emergency department (ED) utilization [1–6], even though EDs are the first point of contact in the acute care system.

Early in the pandemic many patients were treated with experimental therapies including antivirals such as ritonavir/lopinavir, antimalarials such as hydroxychloroquine and antihelmintics such as ivermectin based on anecdotal evidence or inconclusive studies, some of which have been disproven [8–10]. While high-quality randomized rials identified effective therapies and clear indications for their use [11–13], others remain unsupported by high quality evidence [14–16]. Evaluating treatments and resource utilization over time is important to understanding the uptake of evidence-based therapies and their associated patient outcomes.

The Canadian COVID-19 ED Rapid Response Network (CCEDRRN, pronounced “sedrin”) is a national collaboration that harmonized data collection on consecutive COVID-19 cases in EDs across 8 provinces [17, 18]. CCEDRRN’s goal is to generate real-world high-quality observational studies to inform the pandemic response [19, 20]. Our main objective was to describe and compare treatments, acute care utilization, and outcomes of ED patients with COVID-19 across two pandemic waves.

## Methods

### Design and setting

This pan-Canadian observational study enrolled consecutive eligible COVID-19 patients who presented to the EDs of 46 participating acute care hospitals between March 1 and December 31, 2020 [17]. The research ethics boards of participating institutions reviewed and approved the study protocol with a waiver of informed consent for patient enrollment. Patient partners from different areas across the country were engaged from study inception to completion. Study sponsors were not-for profit organizations, and had no role in study design, data collection, analysis, interpretation, or writing of this manuscript. All authors had access to study data and vouch for this manuscript.

### Study population

Research assistants screened institutional or provincial medical microbiology testing lists for nucleic acid amplification tests (NAATs) for severe acute respiratory syndrome coronavirus 2 (SARS-CoV-2) and lists of presenting complaints or discharge diagnoses for consecutive eligible patients [17]. We excluded data from two sites that were unable to initiate data entry in 2020, and two sites that were unable to demonstrate ≥ 99% compliance with patient enrollment to ensure an unbiased sample.

We included COVID-19 patients presenting to the EDs of participating sites who were seen by an emergency physician, and whose medical record review was complete before the data cut (ESM Appendix Fig. 1). We excluded patients tested in the context of an elective admission, those seen directly by a consultant, and those who acquired COVID-19 in-hospital.

### Definitions

We defined confirmed COVID-19 as presenting with COVID-19 symptoms and a positive SARS-CoV-2 NAAT obtained 14 days prior to, or after ED arrival. This allowed us to capture patients diagnosed in the community, and those with early false negative tests. We included patients presenting with COVID-19 symptoms and diagnosed with “confirmed COVID-19" to capture patients who were transferred into CCEDRRN hospitals whose NAAT at the sending site could not be confirmed, and patients who were presumed by treating clinicians to have COVID-19 despite negative NAATs.

We defined repeat COVID infections as cases in whom SARS-CoV-2 was isolated on two ED visits at least 90 days apart based on the longest duration of viral shedding [20–22].

We defined a wave as a period of sustained acceleration followed by a period of sustained deceleration in cases using the World Health Organization (WHO) dashboard for Canada. We allocated patients to the first wave if they presented between March 1 and June 30, 2020, and to the second wave if they presented between July 1 and December 31, 2020.

We defined severe COVID-19 according to WHO criteria [23]. For adults, criteria included an oxygen saturation of < 90% on room air, a respiratory rate > 30 breaths per minute, or signs of severe respiratory distress were documented in the ED.

### Data collection

Trained research assistants abstracted demographic, clinical, treatment, diagnostic and outcome variables from clinical records using standardized forms. We previously evaluated the inter-rater agreement between retrospective chart review and prospectively collected data [17]. We implemented data verification and quality checks to ensure high data quality [17]. Research assistants were unaware of this research protocol at the time of chart abstraction.

We calculated the seven-day moving average incident COVID-19 cases per 100,000 population for every health region [24]. We mapped every patient to the seven-day moving average incident COVID-19 case count of their health region using their postal code of residence and index visit date. We imputed values by modeling reported COVID-19 over time using linear interpolation for the first five weeks of the pandemic, as incident COVID-19 case data were not available for this period (0.1% of values) [24].

### Outcomes

Our primary outcome was in-hospital mortality. Secondary outcomes included treatments administered in hospital, hospital and ICU admissions, ED revisits and readmissions at seven and 30 days.

### Statistical analysis

We summarized patient characteristics, treatments, and outcomes for each wave using descriptive statistics. We assessed wave differences with t-tests or analysis of variance (ANOVA) for continuous variables and chi-square tests for categorical variables. Separate logistic regressions with a random effect for study sites and patients modeled the associations between pandemic wave and outcomes. We considered different adjustments to provide an understanding of the incremental association between factors and pandemic waves: (1) patient (age, sex, comorbidity, tobacco and illicit substance use) and presentation characteristics (arrival mode, arrival from, and disease severity at presentation), and (2) the variables in (1) as well as the seven-day moving average incident COVID-19 cases in the patient’s health region to account for the hospital’s burden of COVID-19 [25]. We fitted continuous variables, such as age and the seven-day moving average incident COVID-19 cases, with restricted cubic splines with three knots into our logistic regression models. We conducted subgroup analyses on patients with severe COVID at presentation, pregnant patients, those reporting unstable housing, and those requiring invasive mechanical ventilation. We provided estimates with 95% confidence intervals (CIs). A cell size restriction policy prohibited us from reporting counts of less than five. A *p* value less than 0.05 was considered statistically significant. We conducted analyses using Stata (Version 16.1, StataCorp, College Station, Texas).

## Results

### Main results

**Fig. 1:**
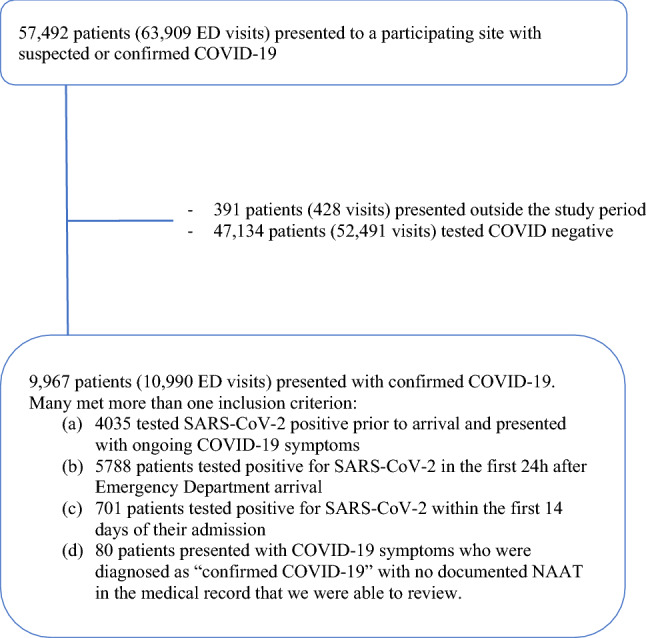
Patient flow diagram

We enrolled 9,967 COVID-19 patients, of whom 3,336 (33.5%) presented in the first and 6,631 (66.5%) in the second wave (Fig. 1). Of these, 3,319 were enrolled in Quebec (33.3%), 2,868 in Alberta (28.8%) and 2,458 in British Columbia (25.6%). In all but 80 (0.8%) patients, a NAAT confirmed the COVID-19 diagnosis. Follow-up time was 30 days for discharged patients and between 30 and 229 days for admitted patients.

### Pandemic waves

Patients presenting to hospitals differed between waves (Table 1 and ESM Appendix Table 1). During the second wave, patients were younger (mean age 53.2 versus 59.4 years old) and with fewer comorbidities compared to the first wave. In the second wave, patients were less likely to arrive from long-term care (5.6% versus 18.3%), report an occupational exposure (2.3% versus 8.7%), travel-related infection (1.9% versus 6.8%) or an institutional exposure (7.5% versus 18.6%). Fewer patients met criteria for severe disease in the second wave (27.7% versus 31.7%).

**Table 1:** Patient and presentation characteristics by pandemic wave

Unique patients (= 9,967)	First wave	Second wave
	(*n* = 3,336)	(*n* = 6,631)
Age (in years) mean (SD)	59.4 (20.7)	53.2 (21.4)
Female (%)	49.7%	49.2%
Pregnant (%)	1.1%	1.2%
Arrival from (%)		
Home	78.6%	89.6%
Long-term care or rehab facility	18.3%	5.6%
Unstable housing*	1.0%	2.1%
Corrections	0.2%	< 0.1%
Inter-facility transfer	1.2%	1.0%
Goals of care (%)		
Full code	77.5%	88.8%
Intermediate goals of care	10.3%	2.8%
Do not resuscitate	10.0%	7.9%
Risk for infection (%)		
Institutional (long-term care, corrections)	19.8%	7.5%
Unknown	15.2%	32.5%
Household contact	12.6%	14.5%
Occupational (healthcare worker)	8.7%	2.3%
Travel	6.8%	1.9%
Comorbidities (%)		
Hypertension	36.0%	27.6%
Diabetes	17.6%	15.9%
Coronary artery disease	8.8%	6.0%
Asthma	7.4%	7.0%
Chronic lung disease, not asthma	6.9%	5.2%
Congestive heart failure	3.9%	3.5%
Active cancer	3.6%	3.2%
Obesity	2.0%	1.9%
Moderate/severe liver disease	0.5%	0.4%
Tobacco use (%)	2.8%	3.9%
Illicit substance use (%)	1.3%	2.7%
Unique ED visits (10,990)	(*n* = 3,679)	(*n* = 7,311)
Arrival by ambulance (%)	48.6%	40.5%
Canadian triage acuity score (%)		
CTAS 1 (resuscitation)	5.1%	3.2%
CTAS 2 (emergent)	28.2%	27.7%
CTAS 3 (urgent)	51.0%	51.4%
CTAS 4 (less urgent)	13.5%	15.7%
CTAS 5 (non-urgent)	1.9%	2.1%
Arrival vital signs, mean (SD)		
Heart rate, beats per min	93.7 (21.5)	93.3 (19.2)
Systolic BP, mm Hg	130.9 (21.6)	130.8 (21.2)
Oxygen saturation, %	95.3 (4.2)	96.0 (3.7)
Respiratory rate, breaths per min	21.7 (6.5)	21.1 (6.5)
Temperature, degrees Celsius	37.3 (0.9)	37.0 (0.9)
Symptoms reported at ED arrival (%)		
Cough	58.5%	52.8%
Dyspnea	52.2%	49.6%
Fever	49.1%	38.6%
General weakness	28.5%	30.0%
Chest pain	24.1%	29.4%
Diarrhea	14.9%	13.7%
Nausea/vomiting	14.2%	18.6%
Headache	13.6%	17.3%
Chills	12.3%	17.6%
Myalgia	12.0%	15.9%
Sore throat	11.1%	12.3%
Altered consciousness	10.5%	7.7%
Dysgeusia/anosmia	3.6%	5.9%
No symptoms	3.4%	3.0%
Pre-ED cardiac arrest	< 0.1%	0.1%
Symptom duration at time of the ED visit**		
Mean (SD) Median (IQR)	6.0 (6.5) 4 (2–8)	5.1 (5.3) 4 (1–7)
WHO severe disease at ED arrival (%)***	31.7%	27.7%

*SD* standard deviation, *CTAS* Canadian Triage Acuity Score, *IQR* interquartile range, *ED* Emergency department

*Unstable housing includes homeless, shelter, single room occupancy

**The denominator for symptom duration is 2823 for wave 1, and 5106 for wave 2

***We defined presentations for severe COVID-19 disease according to WHO age-based criteria. For adults, criteria for severe COVID-19 were met if the patient had an oxygen saturation of < 90% on room air, a respiratory rate > 30 breaths per minute, or signs of severe respiratory distress documented in the ED medical record

Steroids were used more frequently (28.0% versus 9.5%, *p* < 0.0001), and antimalarials (0.3% versus 9.0%, *p* < 0.0001) and antivirals (1.5% versus 6.7%, *p* < 0.0001) less frequently in the second wave (Table 2 and ESM Appendix Table 2). Among admitted patients, steroid use increased to 83.5% in the second wave compared to 16.9% in the first (*p* < 0.001), while antibiotic use remained unchanged (84.3 versus 85.3%, *p* = 0.6). Differences persisted after adjustment for baseline patient characteristics and disease severity (Tables 3, 4). Fewer patients were mechanically ventilated (3.7% versus 7.0%, *p* < 0.0001) in the second wave, which also persisted after adjustment (OR 0.56; 95% CI 0.44–0.71). Patients were intubated at the same time after the onset of symptoms (6.5 versus 6.3 days, *p* = 0.81), but later in their hospital course (3.2 versus 2.0 days, *p* < 0.0001) in the second versus the first wave, and for a shorter duration of time (12.8 versus 16.4 days, *p* = 0.018; ESM Appendix Table 3).

**Table 2:** Acute care utilization and treatments of 9,967 patients, by pandemic wave

	First wave (*n* = 3,336)	Second wave (*n* = 6,631)
Emergency department visits		
One ED visit (%)	90.7%	91.1%
Two ED visits (%)	8.1%	7.9%
Three or more ED visits (%)	1.2%	1.0%
Admissions		
Never admitted (%)	47.0%	61.5%
One admission (%)	51.7%	37.4%
Two admissions (%)	1.2%	1.0%
Three or more admissions (%)	< 0.2%	< 0.1%
Hospital days per admitted patients		
Mean (SD)	15.6 (20.6)	11.6 (12.0)
Median (IQR)	8 (4–19)	8 (4–15)
Admitted to critical care (%)^a^	12.6%	7.7%
Critical care days per critical care admitted pts		
Mean (SD)	15.6 (20.5)	10.5 (11.3)
Median (IQR)	10 (4–19)	6 (3–13)
Medication use (%)		
Steroids	9.5%	28.0%
Antibiotics	48.3%	35.7%
Antivirals	6.7%	1.5%
Anticoagulation (heparin or oral)	39.7%	32.0%
Antimalarials	9.0%	0.3%
Supplemental oxygen (%)	28.6%	16.7%
Most aggressive form of oxygen delivery used (%)		
Mechanical ventilation (%)	7.0%	3.7%
CPAP/BiPAP	0.2%	0.3%
High-flow nasal oxygen	0.5%	0.8%
Simple or non-rebreather facemask	2.6%	1.6%
Nasal prongs	18.4%	10.6%

*ED* Emergency department, *SD* standard deviation, *CC* critical care, *CPAP* continuous positive airway pressure, *BiPAP* Bilevel airway pressure

^a^Includes critical care, high acuity/step down, and operating room (without surgery)

**Table 3:** Adjusted difference in therapy between 9,903 visits in wave 1 and wave 2

Treatments (%)	First wave^b^ (*n* = 2,690)	Second wave (*n* = 7,213)	Adjusted odds ratio^a^ (95% CI)
Mechanical ventilation	166 (6.2)	245 (3.4)	0.56 (0.44–0.71)
Oxygen use	620 (23.1)	1,011 (14.0)	0.93 (0.79–1.01)
Steroid use	201 (7.5)	1,867 (25.9)	7.44 (6.21–8.90)
Antiviral use	181 (6.7)	96 (1.3)	0.16 (0.12–0.22)^c^
Anticoagulant use	931 (34.6)	2,133 (29.8)	1.04 (0.92–1.18)
Antimalarial use	107 (4.0)	22 (0.3)	0.04 (0.01–0.21)

We excluded 960 patients from 4 study sites that did not have enrollment in both waves

^a^Adjusted for age, sex, existing comorbidities (moderate or severe liver disease, hypertension, diabetes, congestive heart failure, coronary artery disease, asthma, chronic lung disease, active cancer, and obesity), WHO severe disease, arrival from, ambulance arrival mode, smoking status, and illicit substance use

^b^Reference category

^c^Did not adjust for moderate or severe liver disease due to collinearity

**Table 4:** Adjusted difference in therapy between 2,986 visits with WHO severe disease on arrival in wave 1 and wave 2

Treatments (%)	First wave^b^ (*n* = 974)	Second wave (*n* = 2,012)	Adjusted odds ratio^a^ (95% CI)
Mechanical ventilation	125 (12.8)	186 (9.2)	0.58 (0.45–0.77)
Oxygen use	442 (45.4)	690 (34.3)	1.06 (0.83–1.34)
Steroid use	120 (13.2)	1,061 (52.7)	9.35 (7.38–11.86)
Antiviral use	94 (9.6)	57 (2.8)	0.24 (0.17–0.34)^c^
Anticoagulant use	495 (50.8)	1,068 (53.1)	1.22 (1.01–1.48)
Antimalarial use	56 (5.7)	9 (0.5)	0.05 (0.02–0.11)^c^

We excluded 960 patients from 4 study sites that did not have enrollment in both waves

^a^Adjusted for age, sex, existing comorbidities (moderate or severe liver disease, hypertension, diabetes, congestive heart failure, coronary artery disease, asthma, chronic lung disease, active cancer, and obesity), arrival from, ambulance arrival mode, smoking status, and illicit substance use

^b^Reference category

^c^Did not adjust for moderate or severe liver disease due to collinearity

A greater proportion of patients were discharged from EDs in the second wave (61.3% versus 47.2%, *p* < 0.0001; Table 5 and ESM Appendix Table 4a). In the second wave a higher proportion of patients revisited the ED within seven days (6.9% versus 5.8%, *p* = 0.025) and were more likely to be admitted to a ward (8.2% versus 6.1%, *p* = 0.008; Table 6 and ESM Appendix Table 4b), but not critical care (ESM Appendix Table 5a). In both waves few patients died in the ED (0.5% versus 0.2%, *p* = 0.016).

**Table 5:** Emergency department visits (*n* = 10,990) by pandemic wave

	First wave (*n* = 3,679)	Second wave (*n* = 7,311)	*p* value
ED visits characteristics			
Index ED visits (%)	90.7%	90.7%	0.97
ED revisits within 7 days (%)	5.8%	6.9%	0.025
ED revisits within 30 days (%)	8.8%	9.0%	0.76
ED disposition (%)			
Admission	49.2%	36.0%	< 0.0001^a^
Home	47.2%	61.3%	
Transfer to LTC, rehabilitation or corrections	1.1%	1.1%	
Transfer to other hospital	1.7%	0.9%	
Left AMA	0.2%	0.3%	
Died in ED	0.5%	0.2%	

*ED* Emergency Department, *LTC* long-term care, *AMA* left against medical advice or without being seen by a physician

^a^ANOVA test for wave differences

**Table 6:** Hospital admissions (*n* = 4,445) by pandemic wave

	First wave (*n* = 1,810)	Second wave (*n* = 2,635)	*p* value
Admission characteristics (%)			
Admission on index ED visit	91.1%	88.4%	0.004
Admission on ED re-visit within 7 days	6.1%	8.2%	0.008
Admission on ED re-visit within 30 days^b^	8.5%	11.0%	0.005
Level of inpatient care (%)			
Ward only	76.7%	80.6%	0.002
Critical care^b^	23.3%	19.4%	
Inpatient trajectory (%)			
From ED to ward	76.7%	80.6%	0.001^a^
From ED to critical care^c^	14.5%	10.7%	
From ED to ward to critical care^c^	8.8%	8.7%	
Timing and length of admissions (%)			
Admitted to ward on index visit	68.7%	69.0%	0.004
Admitted directly to critical care	21.0%	17.3%	
Length of stay in hospital			
Mean, (SD) Median (IQR)	15.6 (21.0) 9 (4–19)	11.7 (12.0) 8 (4–15)	< 0.0001
Length of stay in critical care^c^			
Mean, (SD) Median (IQR)	15.6 (20.5) 10 (4–19)	10.5 (11.3) 6 (3–13)	< 0.0001
Died during hospitalization (%)	19.1%	16.6%	< 0.0001

^a^ANOVA test for wave differences

^b^Includes 7-day readmissions

^c^Includes high acuity/step down, and operating room for ventilation

In the second wave, hospital admissions were shorter (mean 11.7 versus 15.6 days, *p* < 0.0001), yet readmissions after hospital discharge were rare and similar across waves (Table 6 and ESM Appendix Tables 4b and 5b). In the second wave, fewer patients were admitted to critical care (7.7% versus 12.6%, *p* < 0.0001; Table 2, ESM Appendix Table 2) for a shorter duration of time (10.5 versus 15.6 days, *p* < 0.0001; Table 6, ESM Appendix Table 4b). These differences persisted after adjustment for patient characteristics, disease severity, and the seven-day moving average incident COVID-19 cases (Table 7). Crude mortality was lower in the second wave [6.1% versus 8.5%; odds ratio (OR) 0.69, 95% CI 0.59–0.82]. After adjusting for patient characteristics, disease severity, and the seven-day moving average incident COVID-19 cases we observed a trend towards reduced mortality which was not statistically significant (OR 0.7; 95% CI 0.52–1.05).

**Table 7:** Crude and adjusted effect of pandemic period on the outcomes of 9,903 visits

Outcome	First wave^c^ (*n* = 2,690)	Second wave (*n* = 7,213)	Unadjusted odds ratio (95% CI)	Adjusted odds ratio^a^ (95% CI)	Adjusted odds ratio^a^ + GIS^b^ (95% CI)
Primary outcome					
Hospital mortality	229 (8.5)	437 (6.1)	0.69 (0.59–0.82)	0.89 (0.66–1.21)	0.74 (0.52–1.05)
Secondary outcomes					
Admission to hospital	1,312 (48.8)	2,583 (35.8)	0.63 (0.56–0.72)	0.72 (0.64–0.82)	0.72 (0.63–0.84)
Admission to critical care	331 (12.3)	503 (7.0)	0.59 (0.50–0.70)	0.66 (0.55–0.79)	0.71 (0.58–0.87)

Excluded 960 patients from 4 study sites that did not have enrollment in both waves

^a^Adjusted for age, sex, existing comorbidities (moderate or severe liver disease, hypertension, diabetes, congestive heart failure, coronary artery disease, asthma, chronic lung disease, active cancer, and obesity), WHO severe disease, arrival from, ambulance arrival mode, smoking status, and illicit substance use

^b^The 7-day regional COVID-19 incidence refers to the moving average incident COVID-19 case count of the patients’ health region at the time of their Emergency Department visit

^c^Reference category

### Subgroups

During the study period, fewer than five of 9,967 patients (< 0.05%, 95% CI 0.0002–0.0012%) re-visited a participating ED with a NAAT-confirmed re-infection. Among 119 pregnant patients, 28 (23.5%, 95% CI 16.7–32.0%) required admission, fewer than five (< 3.4%, 95% CI 1.2–8.7%) required mechanical ventilation, and none died. Among 176 patients reporting unstable housing (homeless, shelter, or single room occupancy), 50.6% (95% CI 43.2–57.9%) were admitted, and fewer than five (< 5%, 95% CI 0.84–5.93%) died.

## Discussion

### Interpretation of findings

We compared treatments, acute care utilization, and outcomes of COVID-19 patients presenting to EDs between pandemic waves and found differences in patient characteristics that we believe reflected public health measures to protect seniors and reduce travel [26]. We observed rapid uptake of evidence-based therapies and less use of experimental therapies in the second wave. The dramatic increase in steroid use among admitted patients with severe COVID-19 is consistent with its proven indications. We observed substantial decreases in invasive mechanical ventilation and less hospital and critical care utilization over time with no adverse effect on mortality.

### Comparison to previous studies

Administrative database studies observed decreasing mortality during the Spring of 2020, before evidence-based treatments had been identified [6, 27]. While some hypothesized that these observations were the result of improved clinical care as clinicians gained experience treating COVID-19, it is possible that these findings were the result of ascertainment bias and confounding [7]. Testing restrictions during the first wave resulted in only the sickest COVID-19 patients being diagnosed, introducing systematic error in mortality estimates due to decreasing severity of diagnosed cases over time [28]. Administrative database studies were unable to capture respiratory parameters required to adjust for disease severity at presentation [29], resulting in mortality estimates that may be confounded [6]. Finally, during the early pandemic, residents of long-term care were tested more liberally than healthier populations. Oversampling of long-term care residents may have increased the early observed mortality risk due to competing risks [27]. In contrast, our study enrolled consecutive eligible patients through to the end of the first wave reducing ascertainment bias and selection bias, and used detailed clinical data to adjust for baseline differences in disease severity. These methodological differences may explain the observed differences in mortality across studies.

We observed changes to the frequency, initiation, and duration of invasive mechanical ventilation consistent with other studies [30]. Early in the pandemic, non-evidence based recommendations for early endotracheal intubation had been disseminated to reduce disease transmission [31]. We observed less frequent, later and shorter duration of invasive mechanical ventilation in the second wave, consistent with updated airway management guidelines. These changes were associated with reduced hospital and critical care resource utilization and no adverse impacts on mortality. Our results provide real-world evidence that a treatment strategy including reduced use of invasive mechanical ventilation was not associated with harm and may be beneficial.

Uptake of steroids among admitted hypoxic patients after publication of randomized trials and a prospective meta-analysis was rapid [32]. Previously, observed uptake of new evidence into clinical practice has been variable and much slower than what we observed [33]. While we did not collect data about knowledge translation strategies, regional COVID-19 treatment guidelines [34], podcasts with COVID-19 content [35] and other online learning tools [36] were widely shared during the first pandemic year, and may have contributed to rapid knowledge uptake.

### Strengths

Unlike previous studies that were limited to single sites [37–39], we enrolled patients in urban and rural, and academic and non-academic sites across Canada. We captured all COVID-19 patients, including vulnerable patients who are typically unable to provide informed consent. We ascertained the outcomes of all enrolled patients, without censoring at 28 or 30 days, or at the time of analysis, as was commonly done in early studies leading to incomplete outcome ascertainment [14, 29]. For these reasons, we believe we were able to minimize ascertainment and selection bias and are confident of the internal validity of our study. We believe that our sample is representative of COVID-19 patients who presented to Canadian EDs during the study period.

### Limitations

We captured data retrospectively and were limited to what was documented in the medical records. Despite research assistants not being able to support data collection in person in EDs during the first wave of the pandemic, we were able to validate our data collection methods by comparing retrospectively with prospectively collected data [17]. While we were unable to link with genomic data to identify variants of concern [40], circulation of variants was limited during the study period. Finally, we were unable to adjust for differences in patient-level goals of care across pandemic waves.

### Clinical and research implications

Canadian acute care physicians rapidly implemented evolving treatment recommendations based on new evidence or expert advice in 2020. While the observational nature of our study does not allow for causal inferences, our data provide evidence that treatment changes were safe and associated with less acute care resource utilization. The observed reduction in the use of invasive mechanical ventilation was not associated with harm, and may be associated with benefit.

Our work highlights the feasibility of collaborating across Canada to enable timely evaluation of real-world practice changes during a pandemic. Attention to data quality, collection of clinical variables, and patient sampling can supplement and refine lessons learned from more rapidly conducted administrative database studies.

### Conclusion

Our study documents rapid uptake of evidence during the COVID-19 pandemic, both for proven and disproven therapies. We saw increased rates of ED discharges and lower hospital and critical care resource use over time. We saw substantial reductions in mechanical ventilation without increasing mortality. Advances in treatment strategies created health system efficiencies without compromising patient outcomes.

## Supplementary Information

Below is the link to the electronic supplementary material.

Supplementary file1 (DOCX 4474 KB)

Supplementary file2 (PDF 219 KB)
