# Supplementary material for: Treatments, resource utilization, and outcomes of COVID-19 patients presenting to emergency departments across pandemic waves: an observational study by the Canadian COVID-19 Emergency Department Rapid Response Network (CCEDRRN)
Source: CJEM. 2022 Apr 1;24(4):397–407. doi: 10.1007/s43678-022-00275-3 (PMC8972682; doi:10.1007/s43678-022-00275-3)
Supplement: Supplementary file 1 — Supplementary file1 (DOCX 4474 KB) [file 43678_2022_275_MOESM1_ESM.docx]

**Appendix Figure 1. Gantt chart style for enrollment dates per site by pandemic wave**

**
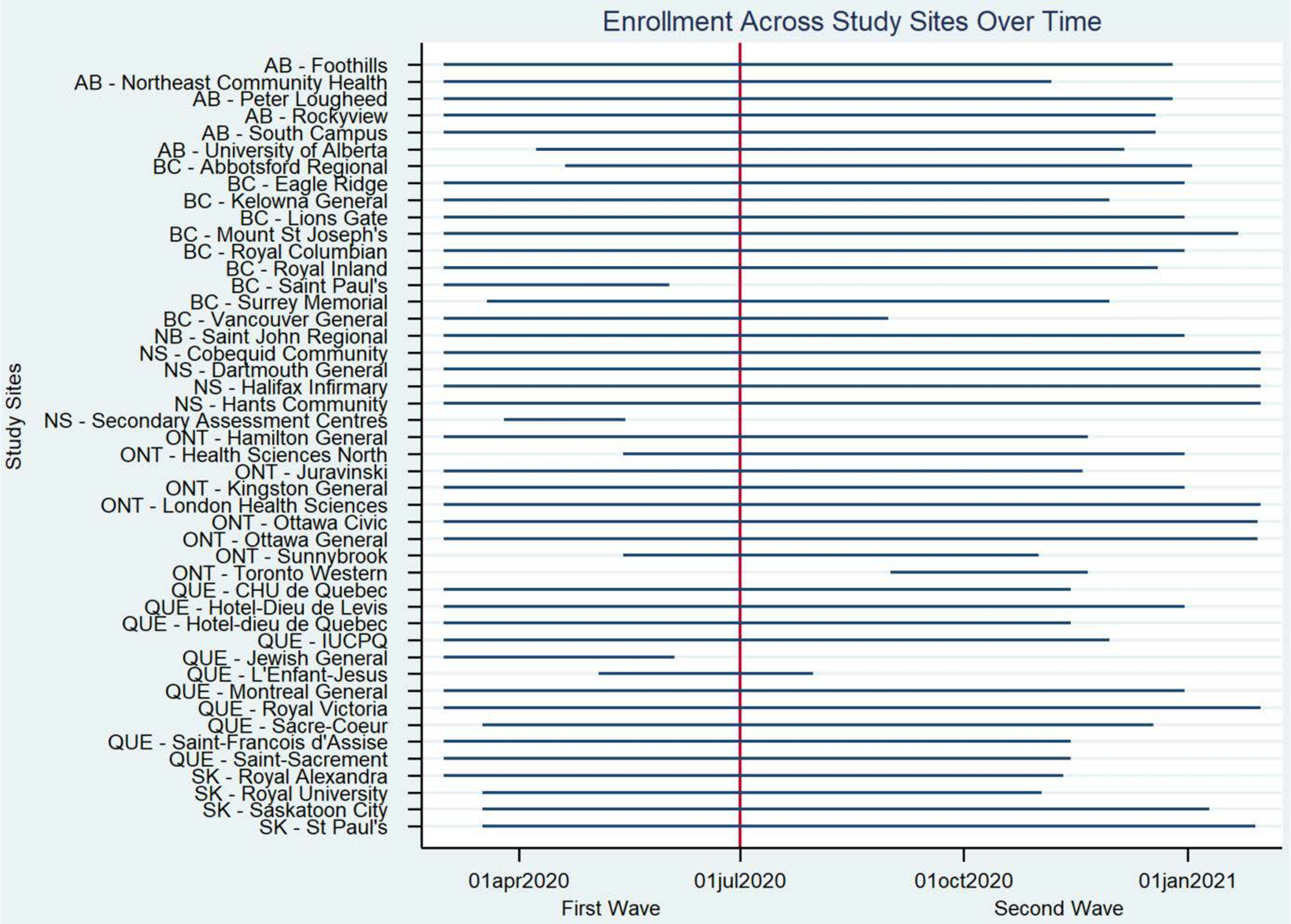
**

NS-Secondary Assessment Centre in NS closed in the first wave; ON-Toronto Western joined CCEDRRN in the second wave. We excluded four of 50 CCEDRRN sites. Two sites started collecting data in 2021, and two sites had incomplete study trackers at the time of the data cut and were thus unable to demonstrate >99% compliance with consecutive eligible patient enrollment.

**Appendix Table 1.** Patient and presentation characteristics by pandemic wave

|  | **First Wave** | **Second Wave** |
| --- | --- | --- |
| **Unique patients (=9,967)** | (n=3,336) | (n=6,631) |
| **Age (in years) mean (SD)** | 59.4 (20.7) | 53.2 (21.4) |
| **Age categories in years (%)** |  |  |
| < 1 | 6 (0.2) | 48 (0.7) |
| 1 – 9 | <5 | 52 (0.8) |
| 10 – 19 | 36 (1.1) | 157 (2.4) |
| 20 – 29 | 242 (7.3) | 780 (11.8) |
| 30 – 39 | 355 (10.6) | 919 (13.9) |
| 40 – 49 | 469 (14.1) | 1,008 (15.2) |
| 50 – 59 | 596 (17.9) | 1,070 (16.1) |
| 60 – 69 | 460 (13.8) | 898 (13.5) |
| 70 – 79 | 452 (13.6) | 821 (12.4) |
| 80 + | 716 (21.5) | 878 (13.2) |
| **Female (%)** | 1,657 (49.7) | 3,262 (49.2) |
| Pregnant (%) | 38 (1.1) | 79 (1.2) |
| **Arrival from (%)** |  |  |
| Home | 2,622 (78.6) | 5,941 (89.6) |
| Long-term care or rehab facility | 609 (18.3) | 373 (5.6) |
| Unstable housing* | 34 (1.0) | 136 (2.1) |
| Corrections | 6 (0.2) | <5 |
| Inter-facility transfer | 39 (1.2) | 66 (1.0) |
| **Goals of care (%)** |  |  |
| Full code | 2,584 (77.5) | 5,886 (88.8) |
| Intermediate goals of care | 344 (10.3) | 187 (2.8) |
| Do not resuscitate | 334 (10.0) | 526 (7.9) |
| **Risk for infection (%)** |  |  |
| Institutional (long-term care, corrections) | 662 (19.8) | 500 (7.5) |
| Unknown | 508 (15.2) | 2,158 (32.5) |
| Household contact | 421 (12.6) | 959 (14.5) |
| Occupational (healthcare worker) | 291 (8.7) | 154 (2.3) |
| Travel | 227 (6.8) | 126 (1.9) |
| **Comorbidities (%)** |  |  |
| Hypertension | 1,200 (36.0) | 1,830 (27.6) |
| Diabetes | 586 (17.6) | 1,051 (15.9) |
| Coronary artery disease | 292 (8.8) | 395 (6.0) |
| Asthma | 247 (7.4) | 463 (7.0) |
| Chronic lung disease, not asthma | 231 (6.9) | 343 (5.2) |
| Congestive heart failure | 129 (3.9) | 229 (3.5) |
| Active cancer | 121 (3.6) | 211 (3.2) |
| Obesity | 65 (2.0) | 126 (1.9) |
| Moderate / Severe liver disease | 15 (0.5) | 24 (0.4) |
| **Tobacco use (%)** | 92 (2.8) | 256 (3.9) |
| **Illicit substance use (%)** | 44 (1.3) | 181 (2.7) |
| **Unique ED visits (10,990)** | (n=3,679) | (n=7,311) |
| **Arrival by ambulance (%)** | 1,786 (48.6) | 2,963 (40.5) |
| **Canadian Triage Acuity Score (%)** | |  |
| CTAS 1 (Resuscitation) | 186 (5.1) | 230 (3.2) |
| CTAS 2 (Emergent) | 1,039 (28.2) | 2,022 (27.7) |
| CTAS 3 (Urgent) | 1,876 (51.0) | 3,755 (51.4) |
| CTAS 4 (Less Urgent) | 498 (13.5) | 1,146 (15.7) |
| CTAS 5 (Non-Urgent) | 71 (1.9) | 150 (2.1) |
| **Arrival vital signs, mean (SD)** |  |  |
| Heart rate, beats per min | 93.7 (21.5) | 93.3 (19.2) |
| Systolic BP, mm Hg | 130.9 (21.6) | 130.8 (21.2) |
| Oxygen saturation, % | 95.3 (4.2) | 96.0 (3.7) |
| Respiratory rate, breaths per min | 21.7 (6.5) | 21.1 (6.5) |
| Temperature, degrees Celsius | 37.3 (0.9) | 37.0 (0.9) |
| **Symptoms reported at ED arrival (%)** |  |  |
| Cough | 2,152 (58.5) | 3,857 (52.8) |
| Dyspnea | 1,922 (52.2) | 3,626 (49.6) |
| Fever | 1,809 (49.1) | 2,822 (38.6) |
| General weakness | 1,049 (28.5) | 2,198 (30.0) |
| Chest pain | 887 (24.1) | 2,153 (29.4) |
| Diarrhea | 547 (14.9) | 1,002 (13.7) |
| Nausea/vomiting | 522 (14.2) | 1,359 (18.6) |
| Headache | 501 (13.6) | 1,266 (17.3) |
| Chills | 451 (12.3) | 1,289 (17.6) |
| Myalgia | 443 (12.0) | 1,163 (15.9) |
| Sore throat | 410 (11.1) | 900 (12.3) |
| Altered consciousness | 387 (10.5) | 561 (7.7) |
| Dysgeusia/anosmia | 132 (3.6) | 432 (5.9) |
| No symptoms | 125 (3.4) | 223 (3.0) |
| Pre-ED cardiac arrest | <5 | 9 (0.1) |
| **Symptom duration at time of the ED visit**** |  |  |
| Mean (SD)  Median (IQR) | 6.0 (6.5)  4 (2 – 8) | 5.1 (5.3)  4 (1 – 7) |
| **WHO Severe Disease at ED arrival (%)***** | 1,156 (31.7) | 2,026 (27.7) |
| SD= standard deviation; CTAS=Canadian Triage Acuity Score; IQR=interquartile range; ED=Emergency Department | | |

* Unstable housing includes homeless, shelter, single room occupancy

** The denominator for symptom duration is 2,823 for wave 1, and 5,106 for wave 2.

*** We defined presentations for severe COVID-19 disease according to WHO age-based criteria. For adults, criteria for severe COVID-19 were met if the patient had an oxygen saturation of <90% on room air, a respiratory rate >30 breaths per minute, or signs of severe respiratory distress documented in the ED medical record.

**Appendix Table 2.** Acute care utilization and treatments of 9,967 patients, by pandemic wave

|  | **First**  **Wave**  (n=3,336) | **Second**  **Wave**  (n=6,631) |
| --- | --- | --- |
| **Emergency department visits** |  |  |
| One ED visit (%) | 3,025 (90.7) | 6,039 (91.1) |
| Two ED visits (%) | 271 (8.1) | 526 (7.9) |
| Three or more ED visits (%) | 40 (1.2) | 66 (1.0) |
| **Admissions** |  |  |
| Never admitted (%) | 1,568 (47.0) | 4,078 (61.5) |
| One admission (%) | 1,724 (51.7) | 2,481 (37.4) |
| Two admissions (%) | 40 (1.2) | 68 (1.0) |
| Three or more admissions (%) | <5 | <5 |
| Hospital days per admitted patients  Mean (SD)  Median (IQR) | 15.6 (20.6)  8 (4 – 19) | 11.6 (12.0)  8 (4 – 15) |
| Admitted to critical care (%)* | 421 (12.6) | 510 (7.7) |
| Critical care days per critical care admitted pts  Mean (SD)  Median (IQR) | 15.6 (20.5)  10 (4 – 19) | 10.5 (11.3)  6 (3 – 13) |
| **Medication use (%)** |  |  |
| Steroids | 316 (9.5) | 1,854 (28.0) |
| Antibiotics | 1,610 (48.3) | 2,368 (35.7) |
| Antivirals | 219 (6.7) | 96 (1.5) |
| Anticoagulation (heparin or oral) | 1,323 (39.7) | 2,119 (32.0) |
| Antimalarials | 300 (9.0) | 21 (0.3) |
| **Supplemental oxygen (%)** | 955 (28.6) | 1,124 (16.7) |
| **Most aggressive form of oxygen delivery used** (%)** |  |  |
| Mechanical ventilation (%) | 232 (7.0) | 247 (3.7) |
| CPAP/BiPAP | 6 (0.2) | 18 (0.3) |
| High-flow nasal oxygen | 16 (0.5) | 52 (0.8) |
| Simple or non-rebreather facemask | 87 (2.6) | 103 (1.6) |
| Nasal prongs | 614 (18.4) | 704 (10.6) |

ED=Emergency Department; SD=standard deviation; CC=critical care; CPAP=Continuous Positive Airway Pressure; BiPAP=Bilevel Airway Pressure

* Includes Critical Care, High Acuity/Step Down, and Operating Room (without surgery)

**Appendix Table 3.** Characteristics of 479 mechanically ventilated patients

|  | **First**  **Wave**  (n=232) | **Second**  **Wave**  (n=247) | **P-value** |
| --- | --- | --- | --- |
| Intubation in ED (%) | 59  (25.4) | 67  (27.1) | 0.67*^t^* |
| Intubation on a ward or in critical care (%) | 173  (74.6) | 180  (72.9) |  |
| Mean days from symptom onset to intubation, (SD) | 6.3  (4.8) | 6.5  (6.1) | 0.81 |
| Days from ED arrival to intubation, mean (SD) | 2.0  (2.7) | 3.2  (4.8) | <0.0001 |
| Days intubated, mean (SD) | 16.4  (15.6) | 12.8  (12.1) | 0.018 |

ED=Emergency Department

*^t^*ANOVA test for wave differences

**Appendix Table 4a**. Emergency Department visits (n=10,990) by pandemic wave

|  | **First**  **Wave**  (n=3,679) | **Second Wave**  (n=7,311) | **P-value** |
| --- | --- | --- | --- |
| **ED visits characteristics** |  |  |  |
| Index ED visits (%) | 3,336 (90.7) | 6,631 (90.7) | 0.97 |
| ED revisits within 7 days (%) | 212 (5.8) | 503 (6.9) | 0.025 |
| ED revisits within 30 days (%) | 323 (8.8) | 655 (9.0) | 0.76 |
| **ED disposition (%)** |  |  |  |
| Admission | 1,810 (49.2) | 2,635 (36.0) | <0.0001*^t^* |
| Home | 1,738 (47.2) | 4,483 (61.3) |  |
| Transfer to LTC, rehabilitation or corrections | 40 (1.1) | 74 (1.1) |  |
| Transfer to other hospital | 62 (1.7) | 64 (0.9) |  |
| Left AMA | 7 (0.2) | 18 (0.3) |  |
| Died in ED | 18 (0.5) | 16 (0.2) |  |

ED= Emergency Department; LTC=long-term care;

AMA=left against medical advice or without being seen by a physician

*^t^*ANOVA test for wave differences

**Appendix Table 4b**. Hospital admissions (n=4,445) by pandemic wave

|  | **First**  **Wave**  (n=1,810) | **Second Wave**  (n=2,635) | **P-value** |
| --- | --- | --- | --- |
| **Admission Characteristics (%)** |  |  |  |
| Admission on index ED visit | 1,649 (91.1) | 2,330 (88.4) | 0.004 |
| Admission on ED re-visit within 7 days | 111 (6.1) | 217 (8.2) | 0.008 |
| Admission on ED re-visit within 30 days* | 153 (8.5) | 290 (11.0) | 0.005 |
| **Level of Inpatient Care (%)** |  |  |  |
| Ward only | 1,388  (76.7) | 2,123  (80.6) | 0.002 |
| Critical care* | 422  (23.3) | 512  (19.4) |  |
| **Inpatient Trajectory (%)** |  |  |  |
| From ED to ward | 1,388 (76.7) | 2,123 (80.6) | 0.001*^t^* |
| From ED to Critical Care** | 262 (14.5) | 283 (10.7) |  |
| From ED to ward to Critical Care** | 160 (8.8) | 229 (8.7) |  |
| **Timing and Length of Admissions (%)** |  |  |  |
| Admitted to ward on index visit | 1,243 (68.7) | 1,817 (69.0) | 0.004 |
| Admitted directly to Critical Care | 380 (21.0) | 456 (17.3) |  |
| Length of stay in hospital  Mean, (SD)  Median (IQR) | 15.6 (21.0)  9 (4 – 19) | 11.7 (12.0)  8 (4 – 15) | <0.0001 |
| Length of stay in Critical Care**  Mean, (SD)  Median (IQR) | 15.6 (20.5)  10 (4 – 19) | 10.5 (11.3)  6 (3 – 13) | <0.0001 |
| **Died during hospitalization (%)** | 346 (19.1) | 436 (16.6) | <0.0001 |
| *Includes 7-day readmissions  **Includes high acuity/step down, and operating room for ventilation | | | |

*^t^*ANOVA test for wave differences

**Appendix Table 5a.** Number of hospital readmissions after index Emergency Department visits from which patients were discharged (n=361)

|  | **First**  **Wave**  (n=124) | **Second Wave**  (n=237) | **P-value** |
| --- | --- | --- | --- |
| **Readmissions after index ED discharges (%)** | | | |
| Readmission to ward within 7 days | 70  (56.5) | 152  (64.1) | 0.15 |
| Readmission to ward within 30 days* | 84  (67.7) | 186  (78.5) | 0.026 |
| Readmission to critical care within 7 days | 32  (25.8) | 35  (14.8) | 0.010 |
| Readmission to critical care within 30* days | 35  (28.2) | 43  (18.1) | 0.027 |

*Includes 7-day readmissions

**Appendix Table 5b.** Number of hospital readmissions after index hospital admissions (n=106)

|  | **First**  **Wave**  (n=38) | **Second Wave**  (n=68) | **P-value** |
| --- | --- | --- | --- |
| **Readmissions after discharge from index admission (%)** | | | |
| Readmission to ward within 7 days | 6  (15.8) | 17  (25.0) | 0.27 |
| Readmission to ward within 30 days* | 26  (68.4) | 44  (64.7) | 0.69 |
| Readmission to critical care within 7 days | <5 | 8  (11.8) | 0.27 |
| Readmission to critical care within 30 days* | 7  (18.4) | 12  (17.7) | 0.92 |

*Includes 7-day readmissions
