# Supplementary material for: Treatments, resource utilization, and outcomes of COVID-19 patients presenting to emergency departments across pandemic waves: an observational study by the Canadian COVID-19 Emergency Department Rapid Response Network (CCEDRRN)
Source: CJEM. 2022 Apr 1;24(4):397–407. doi: 10.1007/s43678-022-00275-3 (PMC8972682; doi:10.1007/s43678-022-00275-3)
Supplement: Supplementary file 2 — Supplementary file2 (PDF 219 KB) [file 43678_2022_275_MOESM2_ESM.pdf]

Canadian
COVID-19 ED
Network

Supplementary Table: Contributors to the Canadian COVID-19 Emergency Department Rapid Response Network

1. Purpose

This supplementary table provides details of the support staff at each of the participating institutions in the Canadian COVID-19 Emergency Department Rapid Response Network. This supplementary document should be attached to each peer-reviewed manuscript after the methods manuscript (M1). The purpose is to ensure research staffs and lead coordinators are appropriately recognized for their contributions to the network.

2. List of Support Staff

Table 1. Network coordinating center staff at the University of British Columbia

Name	Roles	Contributions
Gelareh Ghaderi	Data analyst	Data processing and analysis for manuscripts.
Jeffrey Hau	Data manager	REDCap, data processing and analysis for manuscripts.
Vi Ho	National coordinator	Coordinate with provincial coordinators and training/onboarding of research assistants.
Joe Larkin	Project manager	Project managment
Fiona O'Sullivan	Data analyst	Data processing and analysis for manuscripts.
Serena Small	Research coordinator	Ethics & privacy reviews, data management plan, privacy impact assessment, and qualitative analyses
Amber Cragg	Research manager	Data and manuscript management
Wei Zhao	Data analyst	Data processing and analysis for manuscripts.
Vicky Wu	Data analyst	Data processing and analysis for manuscripts.
Elnaz Bodaghkhani	Research associate	Data and manuscript management

Table 2. Provincial Coordinators

Name	Province	Institutional affiliation	Contributions to CCEDRRN
Corinne DeMone	NS	Dalhousie University, Halifax, Nova Scotia	Research ethics board submission, manages research assistants, data cleaning and quality.
Jacqueline Fraser	NB	Dalhousie University, St. John New Brunswick	Site coordinator as well as research assistant.
Veronique Gélinas	QC	Centre intégré de santé et de services sociaux de Chaudière-Appalaches (Hôtel-Dieu de Lévis site), Lévis	Provincial research coordinator, translation of research material to French, ethics management.

Connie Taylor	ON	Queen's University, Kingston	Coordination of research assistants in Ontario, maintenance of REB applications for the province
Kate Mackenzie	MB	Health Sciences Centre, Winnipeg	Lead RA for the province
Aimee Goss	SK	University of Saskatchewan, Saskatoon	Screens records in Saskatoon, data/extraction and entry, coordinates research assistants.
Hina Walia	AB	University of Calgary, Calgary	Provincial coordinator lead for Alberta, oversight of all Alberta sites.
Rajan Bola	BC	University of British Columbia, Vancouver	Provincial coordinator lead for BC, oversight of all BC sites.

Table 3. Institutional research assistant (RA) leads
Institutional RA leads are responsible for data extraction and integrity, communication with provincial leads.

Name	Province	Institutional affiliation(s)
Corinne DeMone	NS	Dartmouth General Hospital, Cobequid Community Health Centre, Hants Community Hospital<br>Secondary Assessment Centers of the Dartmouth General Hospital, and Halifax Infirmary, Halifax
Jacqueline Fraser	NB	Saint John Regional Hospital, Saint John
Alexandra Nadeau	QC	CHU de Québec Université Laval, Quebec City
Audrey Nolet	QC	Centre intégré de santé et de services sociaux de Chaudière-Appalaches (Hôtel-Dieu de Lévis site), Lévis
Xiaoqing Xue	QC	Jewish General Hospital, Montréal
David Iannuzzi	QC	McGill University Health Center, Montréal
Chantal Lanthier	QC	Hôpital du Sacré-Cœur de Montréal, Montréal
Konika Nirmalanathan	ON	University Health Network, Toronto
Vlad Latiu	ON	Kingston General Hospital, Hotel Dieu Hospital, Kingston
Joanna Yeung	ON	Sunnybrook Health Sciences Center, Toronto
Natasha Clayton	ON	Hamilton General Hospital, Juravinski Hospital, Hamilton
Tom Chen	ON	London Health Sciences Centre, London
Jenna Nichols	ON	Health Sciences North, Sudbury
Kate Mackenzie	MB	Health Sciences Centre, Winnipeg
Aimee Goss	SK	St. Paul's Hospital, Royal University Hospital, Saskatoon City Hospital, Saskatoon
Stacy Ruddell	AB	Foothills Medical Centre, Peter Lougheed Centre, Rockyview General Hospital, South Health Campus, Calgary
Natalie Runham	AB	University of Alberta Hospital, Edmonton

Name	Province	Institutional affiliation(s)
Karlin Su	AB	Royal Alexandra Hospital/Northeast Community Health Center, Edmonton
Josie Kanu	BC	St. Paul's Hospital, Mount Saint Joseph, Vancouver
Bernice Huynh	BC	Abbotsford Regional Hospital and Cancer Center, Abbotsford
Amanda Swirhun	BC	Royal Columbian Hospital, New Westminster
Tracy Taylor	BC	Eagle Ridge Hospital and Health Care Centre, Port Moody
Mai Hayashi	BC	Royal Inland Hospital, Kamloops
Mackenzie Cheyne	BC	Kelowna General Hospital, Kelowna
Sarim Asim	BC	Surrey Memorial Hospital, Surrey
Katherine Lam	BC	Vancouver General Hospital, Vancouver
Kelsey Compagna	BC	Lions Gate Hospital, Vancouver

Table 4. Contributing Study Sites and Investigators

Lead Investigator	Contributing Site / Code	Member Investigators
**Maritime**		
Patrick Fok		
**Nova Scotia**		
Hana Wiemer	Halifax Infirmary/ 902	Patrick Fok
	Dartmouth General Hospital/ 903	Hana Wiemer
	Hants Community Hospital/ 904	Samuel Campbell
	Cobequid Community Health Centre/ 905	Kory Arsenault
	Secondary Assessment Centers of Dartmouth General and Halifax Infirmary/ 908	Tara Dahn
**New Brunswick**		
Kavish Chandra	Saint John Regional Hospital/ 901	Kavish Chandra
**Quebec**		
Patrick Archambault	Hotel-Dieu de Lévis/ 701	Patrick Archambault
	Jewish General Hospital/ 702	Joel Turner
	Centre Hospitalier de l'Université Laval (CHU de Québec)/ 703	Éric Mercier
	L'hôpital Royal Victoria - Royal Victoria Hospital/ 705	Greg Clark
	Hôpital de l'Enfant-Jésus,CHU de Québec/ 706	Éric Mercier
	Hôpital du Saint-Sacrement, CHU de Québec/ 707	Éric Mercier
	Hôpital Saint-François d'Assise, CHU de Québec/ 708	Éric Mercier
	Hôtel-Dieu de Québec,CHU de Québec/ 709	Éric Mercier

Canadian COVID-19 ED Network

	IUCPQ: Institut universitaire de cardiologie et de pneumologie de Québec/ 710	Sébastien Robert
	Hôpital du Sacré-Coeur de Montreal/ 711	Raoul Daoust
**Ontario**		
Laurie Morrison & Steven Brooks	Sunnybrook/ 401	Ivy Cheng
	The Ottawa Hospital - Civic Campus/ 403	Krishan Yadav
	The Ottawa Hospital - General Campus/ 404	Krishan Yadav
	Kingston/Queens/ 406	Steven Brooks
	Hamilton General Hospital/ 407	Michelle Welsford
	Health Science North, Sudbury Ontario/ 408	Rob Ohle
	University Hospital – LHSC/ 409	Justin Yan
	North York General Hospital, Toronto/ 410	Rohit Mohindra
	Victoria Hospital – LHSC/ 412	Justin Yan
	Toronto Western Hospital/ 414	Megan Landes
**Manitoba**		
Tomislav Jelic	Health Sciences Centre/ 307	Tomislav Jelic
**Saskatchewan**		
Phil Davis	Pasqua Hospital, Regina/ 301	Ankit Kapur
	Regina General Hospital, Regina/ 302	Ankit Kapur
	St Paul's Hospital, Saskatoon/ 303	Phil Davis
	Royal University, Saskatoon/ 304	Phil Davis
	Saskatoon City Hospital, Saskatoon/ 305	Phil Davis
**Alberta**		
Andrew McRae	University of Alberta Hospital, Edmonton/ 201	Brian Rowe
	Foothills, Calgary/ 202	Katie Lin
	Rockyview, Calgary/ 203	Andrew McRae
	Peter Lougheed Centre/ 204	Andrew McRae
	South Campus, Calgary/ 205	Stephanie VandenBerg
	Northeast Community Health Centre, Edmonton/ 206	Jake Hayward, Jaspreet Khangura
	Royal Alexandra Hospital, Edmonton/ 306	Jake Hayward, Jaspreet Khangura
**British Columbia**		
Corinne Hohl	Vancouver General Hospital/ 101	Daniel Ting
	Lions Gate Hospital/ 102	Maja Stachura
	Saint Paul's Hospital/ 103	Frank Scheuermeyer

Canadian COVID-19 ED Network

Mount St Joseph's/ 104	Frank Scheuermeyer
Surrey Memorial Hospital/ 105	Balijeet Braar/ Craig Murray
Royal Columbian Hospital/ 106	John Taylor
Abbotsford Regional Hospital/ 107	Ian Martin
Eagle Ridge Hospital/ 108	Sean Wormsbecker
Victoria General Hospital/ 109	Matt Bouchard
Royal Jubilee Hospital/ 110	Matt Bouchard
Nanaimo General Hospital/ 111	Matt Bouchard
Royal Inland Hospital/ 112	Ian Martin
Kelowna General / Hospital/ 115	Lee Graham

It was not possible for us to recruit Members from Newfoundland and Labrador, Northwest Territories, Nunavut, Prince Edward Island and Yukon at the time of the inception of the registry.
